# Manejo en atención primaria de las infecciones de transmisión sexual (II). Lesión ulcerada genital. Vulvovaginitis. Virus del papiloma humano

**DOI:** 10.1016/j.aprim.2024.103012

**Published:** 2024-06-26

**Authors:** Marta Borrull Carrera, Leonardo Barborini Valberde

**Affiliations:** a Facultat de Medicina UB, Universitat de Barcelona, Barcelona, España; b Servei de Medicina Interna, Hospital Universitari Sagrat Cor, Grupo Quirón Salud, Barcelona, España

*Sr. Editor:*

Deseamos aportar algunas contribuciones al muy documentado artículo «Manejo en atención primaria de las infecciones de transmisión sexual (II). Lesión ulcerada genital. Vulvovaginitis. Virus del papiloma humano»^1^.

En primer lugar, sería beneficioso incluir una sección dedicada a la importancia de la educación sexual integral (ESI) en la prevención de las infecciones de transmisión sexual (ITS), incluido el virus del papiloma humano (VPH). La ESI proporciona conocimientos y habilidades necesarias desde una edad temprana para promover una sexualidad saludable y prevenir las ITS.

Además, es fundamental abordar el impacto de la pandemia de COVID-19 en los servicios de atención primaria para las ITS. La interrupción de los servicios de salud durante la pandemia ha afectado el acceso a la atención y ha generado cambios en los patrones de diagnóstico y tratamiento de las ITS. Explorar estas implicaciones ayudaría a comprender mejor los desafíos actuales en la atención a las ITS.

Otro aspecto relevante es el impacto psicosocial de las ITS en la salud mental de los pacientes. Sería útil incluir información sobre cómo el diagnóstico y manejo de las ITS pueden afectar el bienestar emocional de los individuos y la importancia de proporcionar apoyo psicológico dentro de la atención primaria.

Asimismo, es esencial resaltar la importancia de las estrategias de prevención comunitaria, como campañas de concienciación y programas de detección temprana en la comunidad. Estas iniciativas pueden contribuir significativamente a la reducción de las tasas de ITS al promover entornos saludables y seguros para la sexualidad.

Otro aspecto relevante es garantizar un acceso equitativo a la vacunación contra el VPH, especialmente entre grupos vulnerables^2^. Explorar las disparidades en el acceso a la vacunación y proponer medidas para abordar estas inequidades ayudaría a mejorar la cobertura vacunal y prevenir la propagación del VPH^3^.

Finalmente, es importante destacar el papel emergente de la tecnología en la atención a las ITS, incluida la telemedicina y las aplicaciones móviles. Estas herramientas pueden mejorar el acceso a la atención, proporcionar educación y seguimiento a los pacientes, y apoyar la prevención y el control de las ITS de manera innovadora.

En resumen, estas contribuciones adicionales abordan aspectos clave que complementarían el artículo existente, proporcionando una visión más completa y actualizada del manejo de las ITS en atención primaria.

## Financiación

Este trabajo no ha recibido ningún tipo de financiación.
